# Searching for Meaning in Chaos: Viktor Frankl’s Story

**DOI:** 10.5964/ejop.5439

**Published:** 2021-08-31

**Authors:** Hanan Bushkin, Roelf van Niekerk, Louise Stroud

**Affiliations:** 1 The Anxiety and Trauma Clinic, Johannesburg, South Africa; 2 Department of Industrial and Organisational Psychology, Nelson Mandela University, Port Elizabeth, South Africa; 3 Department of Psychology, Nelson Mandela University, Port Elizabeth, South Africa; University of Johannesburg, Johannesburg, South Africa

**Keywords:** Viktor Frankl, existentialism, Holocaust, noö-dynamics, psychobiography

## Abstract

The existential psychiatrist Viktor Frankl (1905–1997) lived an extraordinary life. He witnessed and experienced acts of anti-Semitism, persecution, brutality, physical abuse, malnutrition, and emotional humiliation. Ironically, through these experiences, the loss of dignity and the loss of the lives of his wife, parents and brother, his philosophy of human nature, namely, that the search for meaning is the drive behind human behaviour, was moulded. Frankl formulated the basis of his existential approach to psychological practice before World War II (WWII). However, his experiences in the concentration camps confirmed his view that it is through a search for meaning and purpose in life that individuals can endure hardship and suffering. In a sense, Frank’s theory was tested in a dramatic way by the tragedies of his life. Following WWII, Frankl shaped modern psychological thinking by lecturing at more than 200 universities, authoring 40 books published in 50 languages and receiving 29 honorary doctorates. His ideas and experiences related to the search for meaning influenced theorists, practitioners, researchers, and lay people around the world. This study focuses specifically on the period between 1942 and 1945. The aim is to explore Frankl’s search for meaning within an unpredictable, life-threatening, and chaotic context through the lens of his concept of noö-dynamics.

Viktor Emil Frankl (26 March 1905–2 September 1997) was an Austrian psychiatrist and neurologist, a Holocaust survivor, and the founder of logotherapy—a school of therapy centred around meaning creation, considered the Third Viennese School of Psychotherapy. A central tenet of Frankl’s theory is the concept of noö-dynamics (Frankl, 2014) which helps to explain the relationship between Frankl’s chaotic external world and his search for meaning within the chaos. This study aims to explore how Frankl made sense of his world and ultimately how he found meaning during his time in the concentration camps, between 1942 and 1945. More specifically, this psychobiographical case study explores—through the lens of Frankl’s concept of noö-dynamics—the strategies he employed in the concentration camps to find purpose and create meaning.

## The Life of Viktor Frankl

Frankl was born on the 26th of March 1905 in Vienna in the Jewish area of Leopoldstadt where he personally witnessed and experienced daily acts of anti-Semitism and persecution (Frankl, 2000). In 1930 Frankl received a medical degree from the University of Vienna, and was put in charge of the hospital ward in Vienna for the treatment of females who had attempted suicide (Redsand, 2006). In 1937 Frankl established a private practice in neurology and psychiatry in his sister’s living room (Redsand, 2006). During World War II in September 1942, Frankl and his wife, Tilly were deported to Theresienstadt concentration camp. This was the first of four different camps where Frankl experienced physical abuse, malnutrition, emotional humiliation and torture (Klingberg, 2001; Redsand, 2006). During his time in the concentration camps, Frankl experienced the loss of his wife and parents, and perhaps most significantly, the loss of hope, dignity and meaning, which ultimately moulded his philosophy of human nature (Frankl, 2006; Klingberg, 2001). He was liberated from Türkheim concentration camp in 1945 after spending two and a half years in four concentration camps. A year later Frankl wrote his most distinguished book, *Man’s search for meaning* (Frankl, 1959), which chronicled his experiences in the concentration camps and proposed the foundation of his theory (Southwick, Gilmartin, McDonough, & Morrissey, 2006).

After the war Frankl was appointed as Chief of Neurology at the Vienna Policlinic Hospital. Here he met his second wife, Elly Schwindt (Klingberg, 2001; Redsand, 2006). They were married in 1947 and together taught Frankl’s theory and philosophy (Redsand, 2006). Frankl’s contribution to the academic world has been recognised by major institutions throughout the globe (Graber, 2004; Redsand, 2006). He became blind at the age of 85, and died in 1997 at the age of 92.

## Frankl’s Existential Theory

Frankl’s philosophical background is grounded in existentialism and his theory has been placed in the tradition of existential philosophy (Klingberg, 2001; Pytell, 2015). He described a situation at the age of 13 that would become a central tenet of his theory (Frankl, 2000; Klingberg, 2001; Pytell, 2015; Redsand, 2006). When a teacher told Frankl’s class that life is processes of combustion and oxidation, Frankl asked: “Professor Fritz, if this is the case, what meaning then does life have?” (Redsand, 2006, p. 18). Later Frankl described reductionism “as today’s nihilism” (Frankl, 2000, p. 60). Frankl believed that reductionism failed to grasp the uniqueness of humanness by describing human beings as mere machines, as opposed to possessing the ability to transcend beyond their unique physicality (Frankl, 1988, 2006, 2014).

Frankl (2010, 2011, 2014) maintained that the search for meaning is not a secondary thought process to instincts, but rather the primary motivation in life. His theory (2010, 2011, 2012, 2014) highlighted the need to acquire the tools necessary to find meaning, rather than to view a person as a two-dimensional machine with separate parts. A person’s ability to transcend their environment was a central component of Frankl’s existential theory.

Frankl (2004, 2006, 2014) stated that the individual is the only one to decide about the meaning of their life and that the individual has to take responsibility for creating and deciding its unique meaning. Furthermore, the ability to decide the meaning of a situation has the power to create a positive outcome from the worst of situations, as Frankl (2000) explained:

I can see beyond the misery of the situation to the potential for discovering a meaning behind it, and thus to turn an apparently meaningless suffering into a genuine human achievement. I am convinced that, in the final analysis, there is no situation that does not contain within it the seed of meaning. To a great extent, this conviction is the basis of Logotherapy. (p. 53)

### Noö-Dynamics

Frankl (2006) asserted that mental well-being is not about achieving emotional equilibrium, but rather “the existential dynamics in a polar field of tension where one pole is represented by the meaning that is to be fulfilled and the other pole by the man who has to fulfil it” (p. 110). This is a foundational concept of Frankl’s existential theory, which is based on a person’s drive to achieve purpose in life. Frankl (2004) referred to this tension between a person’s end goal and where a person is currently as noö-dynamics. The term noödynamics is derived from noetics which was a central feature of the Austrian philosophical-psychological tradition to which Frankl was a part. The origin of the term stems from the Greek word noös meaning mind or spirit (Hatt, 1965). Hence, the noölogical dimension according to Frankl refers to the uniquely human experience of transcending one’s environment and entering into the dimension of noetic phenomena (or the noölogical dimension; Frankl, 1988). According to Frankl (2004), human beings should aim to create this tension in order to re-orientate themselves towards their meaning in life. This constant tension provides a person with a sense of drive and purpose (Frankl, 2006, 2014). Frankl stated that working towards a sense of emotional homeostasis is mentally healthy and that tension aroused by a goal that needs to be fulfilled is what makes a person live in this world with purpose. Frankl (2004), therefore, maintained that noö-dynamics is a healthy state for a person to be in and while a state of emotional homeostasis is naturally comforting, noö-dynamics is what one should aim to create in one’s life.

## Method

The present study on the life of Frankl may be described as a longitudinal life history study with a qualitative-morphogenic, idiographic-morphogenic research design (Burnell, 2013; Yin, 2013). The research design may be defined further as a longitudinal, single-case, psychobiographical study (Ferrer & Ponterotto, 2020; Fouché, 1999; Fouché & Van Niekerk, 2010; Ndoro & Van Niekerk, 2019; Van Niekerk, Prenter, & Fouché, 2019), which portrays an individual’s life through the use of evidence, theory and interpretation (Du Plessis, 2017; Du Plessis & Du Plessis, 2018; Mayer, Van Niekerk, & Fouché, 2020; Ndoro & Van Niekerk, 2019; Schultz, 2005). Psychobiographical research is simply the construction of a subject’s lived experiences through the application of psychological theory and involves the comprehensive application of biographical information with the aim of illuminating patterns in thinking, feelings and behaviours in extraordinary individuals (Du Plessis & Du Plessis, 2018; Ferrer & Ponterotto, 2020; Fouché & Van Niekerk, 2010; Mayer et al., 2020; Ponterotto, 2014, 2018; Prenter, Van Niekerk, & Fouché, 2019; Van Niekerk et al., 2019).

This qualitative psychobiographical study can also be described as both exploratory-descriptive and descriptive-dialogic in nature. The exploratory-descriptive nature refers to the nature of exploration of Frankl’s process of creating meaning through the lens of his concept of noö-dynamics, while the descriptive-dialogic nature of this approach allowed for the informal assessment of the same psychological concept to be applied to Frankl’s experiences in the concentration camps.

### Sampling

A non-probability sampling procedure, purposive sampling, was employed in the selection of Frankl as the psychobiographical subject. In purposive sampling, the researcher’s judgement is used to determine the characteristic attributes desired and to ensure that the data collected and analysed are in-depth (Strydom & Delport, 2005). Frankl was selected for this study based on his unique, significant and interesting life.

### Data Collection and Analysis

Multiple sources of data were collected and reviewed. The researchers searched for publicly available material related to the historical period in which Frankl lived. The material included primary data such as books written by Frankl, as well as secondary data, which included materials produced by others about Frankl’s life and contributions. The sources of information collected were aligned with the primary aim of this study and included an autobiography, biographies, published books and articles by Frankl and about Frankl, transcribed interviews and lectures presented by Frankl.

Yin (2013) proposed two strategies that should be employed by researchers, namely data analysis that is guided by objective theoretical approaches and the strategy of case description. The first strategy refers to how the researcher relies on the theoretical approaches to identify and select the data to be used in the collection and analysis process. This selection is achieved through the researcher asking questions that will provide insight into the objectives of the study, as well as the theoretical approaches used (Fouché, 1999). The second strategy entails the development of a descriptive framework to organise and integrate case information (Yin, 2013). According to Fouché (1999), the researcher should achieve this through the development of a conceptual matrix that would guide the data extraction and categorisation. Both Alexander’s model (Alexander, 1990), as well as Du Plessis’s 12-step process (Du Plessis, 2017) was used for this purpose.

### Qualitative Research Criteria

In order to ensure a satisfactory qualitative research quality, the researchers applied qualitative criteria, more specifically referring to trustworthiness, credibility, dependability, transferability and conformability (Yin, 2013). In addition, a process of triangulation of theories, data and method was applied (Tindall, 1999).

### Ethical Considerations

Ethical clearance was provided by the Nelson Mandela University in Port Elizabeth, South Africa. The researchers chose to study a long-deceased individual, which eliminated the need to receive consent from the subject. However, the researchers obtained written consent from the Viktor Frankl institute for conducting the study. In addition, the researchers strictly followed the recommendations made by Elms (1994) and also complied with the general ethical guidelines as stipulated by the Health Professions Council of South Africa.

Finally, the study was written with consciousness regarding the sensitive personal information used and the findings made. This was done with the intent to not cause embarrassment to Frankl’s family.

## Frankl’s Search for Meaning in Chaos

Frankl’s concept of noö-dynamics and homeostasis (Frankl, 1988, 2004) is a central idea that explains the push-pull relationship between Frankl’s inner and outer chaotic world and his subsequent need to create meaning within the chaos. Frankl’s struggle to understand his life, his need to find answers and to establish the meaning in such a struggle permeated throughout his life, and more specifically during his time in the concentration camps between 1942 and 1945. Frankl wrote, discussed, and taught many principles and strategies to help others find meaning in their lives. The researchers have observed, collated, and highlighted at least eight specific strategies which Frankl had utilised in order to create meaning within his chaotic environment. More specifically, the creation of meaning through: 1) creative pursuits, 2) servicing others, 3) contradictory experiences, 4) the commitment to a decision, 5) spiritual connection, 6) perceiving meaningless tasks through a meaningful lens, 7) creating and chasing goals, and lastly 8) maintaining an unconditional attitude of strength. More specific examples of how Frankl utilised these strategies are highlighted below.

Over a period of two and a half years, Frankl had lived in four concentration camps: Theresienstadt, Auschwitz, Dachau and Türkheim. Life at Theresienstadt concentration camp was harsh and filled with daily suffering, although Frankl recalled how the Jews in the camp attempted to keep their lives meaningful and entertaining (Frankl, 2000, 2012). Children and artists painted, actors performed for the crowds, musicians played the music that uplifted people and scholars gave lectures (Adler, 2017). Frankl found that witnessing and participating in creative pursuits provided him with meaning and purpose in a place which was intent on creating suffering. For Frankl, engagement with creative outlets provided him with meaningful distraction, which ultimately allowed him to transcend the chaos of his environment and escape through engagement with something meaningful. In essence, the daily suffering had created a sense of noö-dynamics, and the engagement in creative pursuits helped Frankl to achieve a sense of homeostasis.

Frankl volunteered to give public talks on different topics related to medicine and psychology, for example, sleep disturbances, the psychology of Alpinism (mountain climbing), medical ministry, and psychotherapy (Klingberg, 2001). Furthermore, as he had organised in Vienna, Frankl was in charge of a team of like-minded individuals who participated in and ran suicide-prevention programs that helped prisoners adjust to life in Theresienstadt (Klingberg, 2001; Redsand, 2006). Frankl and his team often helped depressed and suicidal prisoners with logotherapeutic techniques that aimed at assisting them in finding meaning and reasons to live despite their living conditions (Frankl, 2000). Frankl found that pursuing activities for the service of others allowed him to transcend the suffering of his environment. Such pursuits had changed Frankl’s focus from himself to focusing on others. This change in focus had resulted in living a life of meaning within an environment that was designed to take meaning away. The daily life in the camp had created a sense of suffering or noö-dynamics for Frankl, and his engagement in activities that contributed to others, provided him with meaning and purpose, ultimately at times homeostasis. The psychological need to create homeostasis had once again provided Frankl with a pursuit for meaning.

Another example of Frankl finding meaning in the pursuit of serving others was when after 5 months of hard labour at Kaufering, which was one of the sub camps in Dachau (on 5 March 1945), he was approached by the chief doctor of Kaufering, a Hungarian who felt favourable towards Frankl (Frankl, 2000). Frankl was asked if he had wanted to head towards Türkheim (also known as Kaufering IV), where he would work as a doctor. Frankl was sceptical of the offer as he did not trust the real intention of the officials, especially since there was a chance that he would be tricked into going to a death camp. Frankl decided to go as he felt that if he spent the last remaining moments of his life caring for sick prisoners, at least his suffering, life and death would have some meaning. Such act of engagement in a meaningful pursuit, allowed Frankl to gain meaning in the chaotic and challenging circumstances. The noö-dynamics created through the unpredictable environment was given meaning through the pursuit of helping others, offering them hope and emotional comfort (Frankl, 2000).

The Nazis humiliated and tortured the people of Theresienstadt daily (Klingberg, 2001). Frankl recalled that one day he was called to the Gestapo run police prison, where a SS officer ordered him to fill a bucket with water, run to a compost pile and pour the water on top of the pile. The pile was higher than Frankl in length and when he could not reach the top of the pile to pour water over it, the SS officer beat him and ordered him to repeat this exercise for hours. Eventually, Frankl was dragged back to his quarters with 32 injuries (Frankl, 2000). Tilly cleaned his wounds and in an attempt to lift his spirit, she took him to a jazz concert that evening. The contrast provided Frankl with a fascinating insight. He described it as follows: “The contrast between the indescribable torture of the morning and the jazz in the evening was typical of our existence—with all its contradictions of beauty and hideousness, humanity and inhumanity” (Redsand, 2006, p. 65). Frankl once again found meaning in engaging with creative pursuits, but also through the contradiction of his experience. The experience of suffering had created a sense of noö-dynamics, and through the engagement with meaningful activities such as art, culture, and music had created a relief or distraction. In addition, the contradiction between suffering and meaningfulness heightened the feelings of noö-dynamics which ultimately created a greater sense of purpose.

Frankl had endured a total of four separate selections, each one carrying the anxiety of an unknown fate, which he acknowledged taught him to resign to the decisions that were not under his control and “to let fate take its course” (Frankl, 1961, p. 54). In the fourth selection, Frankl was selected for labour in the Dachau camp and was loaded with another group onto a freight train. Frankl had no idea where the train was headed and he and the rest of the prisoners tried to guess the direction (Klingberg, 2001). The train was heading west and Frankl feared that they were heading for Mauthausen, a camp so notorious for torture, that it was feared even amongst the prisoners in Auschwitz. Frankl and the rest of the prisoners on the train felt a sense of relief when the train swerved away from Mauthausen onto a track that led to Dachau in Southern Germany. Frankl recalled that it was a joyous moment for all the prisoners, later emphasising that the size of human suffering is relative and that a trivial experience can cause the most amount of joy (Frankl, 2000). For the prisoners at that moment realising that they were not going to be sent to Mauthausen camp but were instead being sent to Dachau was something to celebrate, even though being sent to Dachau was hardly a cause for celebration (Frankl, 2000; Klingberg, 2001). Frankl had discovered for himself that meaning is constructed through the contradiction of one’s experiences. For Frankl, the bigger the gap between one’s current position and one’s goal, vision or the position of where one wants to be, the greater the sense of noö-dynamics. Once the difference lessens, the experience and the emotion felt is joy.

Frankl also found meaning in enjoying small pleasures (Frankl, 2012). Frankl recalled sleeping in his clothes at night because the wintertime was unbearably cold. He remembered the moment of heat as he lay on the loose earth and urinated in his clothing, which gave him immense pleasure (Frankl, 2000). The same pleasurable moment was when he was standing in soup lines, enjoying the warm sensation of urinating in his clothes. At that moment, it felt like “sipping a hot tea” (Klingberg, 2001, p. 2632/6819). Frankl found that meaning was relative, and is constructed through the contradiction of one’s experience.

The conditions in the Ghetto took its toll on Frankl’s father, Gabriel. He was placed in the same barracks where Viktor had lived and worked (Klingberg, 2001). Six months into their stay in Theresienstadt, Gabriel died of starvation and pneumonia at the age of 81 years (Frankl, 2000). Frankl reported that his father’s death left him feeling at peace because he knew that he had done all he could in order to protect his father by the choice that he made to stay in Vienna. Regarding his choice Frankl recalled:

I kissed him and left. I knew I would not see him alive again. But I had the most wonderful feeling one can imagine. I had done what I could do. I had stayed in Vienna because of my parents and now I had accompanied father to the threshold and had spared him the unnecessary agony of death. (Klingberg, 2001, p. 2289/6819)

Frankl had found meaning through committing to a decision without considering the alternative. This commitment had allowed Frankl to not place value on alternative positions and hence live fully engaged with the decision made. Such act of committing to a position had created psychological closure or homeostasis, as it helped Frankl counterbalance the loss of his father with the sacrifice that he made by staying with his parents. Ultimately providing his sense of loss with meaning.

Another example of Frankl finding meaning through his commitment to a position or a choice was when he was at Auschwitz. Frankl and the rest of the prisoners in the line were ordered to go to the cleansing station, where they were instructed to toss their valuables onto blankets on the ground. Frankl rebelled and hid his two most valuable items in his possessions, with the first being his manuscript about logotherapy, which he had hoped would be his legacy and the second being the Donauland Alpine pin he had earned as a climbing guide. He hid both in his jacket, which he had also hoped to keep. Unfortunately, Frankl was ordered to throw his clothes into a pile and with it, his most treasured possessions (Frankl, 2000). Frankl’s commitment to a decision to hold onto his meaningful objects had created the counterbalance to the psychological chaos (noö-dynamics) which was created in his environment.

At Auschwitz, Frankl’s body hair was shaved off and he was ordered to shower with the other prisoners (Klingberg, 2001). The prisoners had heard stories about other prisoners being ordered to get ready for the showers, given soap, only to realise that they were in the gas chamber when the door closed behind them. Frankl was relieved when he realised that real water poured out of the showerheads (Frankl, 2000). Frankl emerged from the showers and was ordered to pick clothes from a pile of clothes that lay on the floor. These clothes belonged to prisoners who were murdered in the gas chambers. Frankl picked a thin, torn coat from the pile and found a scrap of paper in the pocket (Redsand, 2006). It was a torn page from the Jewish prayer book and on it was written the Shema Yisrael, the prayer Frankl had heard his father say every day as a young boy. The prayer translated from Hebrew said: “Hear, O Israel, the Lord our God, the Lord is One God; and you shall love the Lord our God with all your heart and with all your soul and with all your strength” (Redsand, 2006, p. 70). Frankl later wrote that this prayer and his connection to a spiritual realm was a “challenge to me to live what I had written, to practice what I had preached” (p. 70). The chaos which Frankl was living in had created a sense of noö-dynamics which motivated him to search for a meaningful reason to continue living through the suffering. Frankl had found the words of the prayer and spiritual connection as a message that had once again created a stronger sense of homeostasis and purpose.

Another example of Frankl connecting to a spiritual dimension in order to find inner resiliency and ultimately meaning, was when he recalled shovelling snow and struggling to find meaning for such great suffering. Frankl questioned the purpose of this type of life, and at that moment, he “heard a victorious Yes” (Redsand, 2006, p. 75), looked up and a light from a farmhouse in the distance went on. The light turning on coincided with his inner voice resounding that life does have a purpose, which helped reignite Frankl’s desire to continue living (Frankl, 2000). It was at that moment that Frankl decided to pursue the rewriting of the book he had lost at Auschwitz, which would later be titled *The Doctor and the Soul.* Frankl acknowledged that the connection to a spiritual dimension provided him with a sense of purpose and ultimately kept him alive (Frankl, 2000; Klingberg, 2001).

Frankl also created meaning by perceiving a meaningless task through a meaningful lens. For example, when the prisoners reached Dachau and were sent to one of its subcamps, known as Kaufering III. At this camp, while enclosed by barbed wire, there were no gas chambers, ovens and no crematoria at this camp (Klingberg, 2001). In the Kaufering III camp, prisoners were assigned to build concrete bunkers and railway supply embankments. Under the physically challenging conditions, prisoners fell ill quickly after arriving and in September and October 1944, 1,322 ill prisoners were selected to be deported to Auschwitz to be gassed (Redsand, 2006). Frankl was assigned to be a manual labourer, working on railroads, digging ditches and building new camps. Frankl later acknowledged that his mountain climbing experience had helped him to survive physically. Frankl recalled loading sick prisoners onto a wagon, while another prisoner said to him: “Frankl, I see from how you are proceeding that you have a way of conserving your energy when you are not using it to do something, like an Alpine climbing guide” (Redsand, 2006, p. 74). Frankl, found meaning in a task that seemed meaningless, by assigning meaningful pursuits to meaningless activities, more specifically in this example was the activity of mountain climbing which provided him with so much joy.

For survival, Frankl recalled that the prisoners focused their attention on their dreams and fantasies and on relatively small goals, such as attaining food, getting better clothes to wear or just avoiding punishment (Frankl, 2012). However, for Frankl, it was not only the small goals that helped him cope emotionally, but focusing on the future and future goals that provided him with the most meaning (Frankl, 2000). This realisation which would become one of the central principles of his existential theory came up during what Frankl called “the endless little problem of our miserable life” (Redsand, 2006, p. 75). During his suffering, while strategising on how to get bread with his meal or how to get a piece of wire to tie his shoe or how to get the Capo (a prisoner with extra privileges who acted as a foreman) to give him a safer job, Frankl had this insight and he daydreamed about his future, about standing on a platform of a well-lit lecture hall, lecturing about the psychology of the concentration camp. This future dream would become his goal and Frankl realised that focusing on that future goal, rather than on the unchangeable situation, served as his coping and survival mechanism. Frankl wrote that at that moment, he “succeeded somehow in rising above the situation, above the suffering of the moment” (Redsand, 2006, p. 75).

Another example of Frankl’s approach to chasing goals as means of attaining meaning in chaos was when he would council suicidal prisoners. For the prisoners, life was filled with daily suffering and ending one’s life was an enticing option (Langer, 1982). Therefore, a strict rule to be adhered to by the prisoners was to not interfere with a man in the process of committing suicide (Frankl, 2000). Many times, Frankl would approach a suicidal prisoner and attempted to help the person discover a goal to live for, something unique to that person that was aligned with his or her personalised values. Whether it be to live for a child or complete some project which they had begun, whatever the reason, Frankl would attempt to help the person look for it. Frankl recalled being asked by a warden to speak to the group of prisoners and offer words of encouragement. Since Frankl also experienced a sense of hope, the topic of his talk to his fellow prisoners was about hope for the future, loved ones and about chasing goals that were unfinished that needed to be finished. Frankl spoke about finding meaning despite their situation and quoted Friedrich Nietzsche: “That which does not kill me makes me stronger” (Redsand, 2006, p. 76). Frankl recalled that he had offered his fellow hopeless men hope for the future by getting them to focus on meaningful goals to pursue (Frankl, 2000).

Frankl noticed something interesting in the camp; often, it was not the physically strong men who emotionally survived (Frankl, 2006). Frankl questioned the reasons behind such observation and realised that emotional and psychological survival in an unchangeable environment is often not dependent on physical strength, but rather inner strength (Frankl, 2006, 2012). Frankl found that maintaining an unconditional attitude of strength despite one’s environment was a significant ingredient that allowed the prisoners to develop emotional resiliency in their chaotic environment.

## Discussion

Frankl’s theory places much emphasis on strategies and principles of how to create meaning in one’s unchangeable circumstance and environment. More specifically, Frankl (2014) proposed that the creation and discovery of meaning in one’s life can be achieved through creative pursuits, the experience of love, and through an attitudinal value. Through such methods, Frankl maintained that a person can overcome the anxiety surrounding one’s own finite life, suffering, and guilt. The push-pull relationship or the emotional tension created within the individual when chasing one’s goals and the eventual relief of achieving the desired results was a common theme throughout Frankl’s experiences in the camp and his methods of creating meaning in his experiences. More specifically, Frankl created meaning in his experiences through: 1) creative pursuits, 2) servicing others, 3) the contradiction of experiences, 4) the commitment to a decision, 5) spiritual connection, 6) perceiving meaningless tasks through a meaningful lens, 7) creating and chasing goals, and lastly 8) maintaining an unconditional attitude of strength.

Whilst the researchers found Frankl’s theory useful in exploring and describing his own search for meaning within his chaotic environment, Frankl’s theory has some limitations which will be highlighted for the reader in order to provide a more robust context and understanding. Firstly, Frankl’s existential theory has been criticised for its simplistic views of human drive and motivation. More specifically, Frankl’s theory oversimplifies the complexities of what motivates human beings to search for meaning (Pytell, 2015; Tengan, 1999). Secondly, Tengan (1999) criticised Frankl’s existential theory on the basis that his theory is not comprehensive enough. More specifically, Tengan believed that Frankl’s notion of freedom of will (Frankl, 1988) is simplistic. However, the researchers maintained that this criticism should not detract from Frankl’s emphasis on the importance of individual will and responsibility, which he had exercised when attaining meaning in his life. The researchers explored and described Frankl’s life and thematic strategies for attaining meaning through the lens of his key theoretical concept of his existential theory (Frankl, 2014). Whilst the study offered a unique perspective on Frankl’s pursuit for meaning during his time in the concentration camps between 1942 and 1945, the researchers recommends that future researchers explore the underlying psychological reasons as to why Frankl was driven to find meaning in an unchangeable environment.

Whilst the researchers maintain that the study has been successful in terms of the achievement of its aims, future research should consider Frankl’s methods of creating meaning in his earlier development. Such research could possibly shed light on additional findings and understanding of Frankl’s methods of creating meaning in other environments, situations, and development.
